# Correspondence

**Published:** 1902-01

**Authors:** 

**Affiliations:** Waycross, Ga.; Waycross, Ga.


### CORRESPONDENCE.

---

WAYCROSS, GA., Nov. 5, 1901.

DEAR DOCTOR: Following out the suggestions of a number of Southern physicians interested in the use ot Animal Therapy, we deem it advisable to organize a Tri-State (Georgia, Florida and South Carolina, or Georgia, Florida and Alabama) Animal Cellular Therapy Association for the convenience of those physicians living in the far South, as it is almost an impossibility for them to attend the meetings of the American Animal Therapy Association.

We want your advice concerning such an organization, place and time of meeting. We would suggest Charleston, S. C., or Atlanta, Ga., as suitable places, and December, 1901, or January or February, 1902, a good time. The Exposition will be open at Charleston, which in itself will be a great attraction. Atlanta will welcome us.

Please let us hear from you at once.

Yours very truly,

DRS. WALKER AND IZLAR.

[We would like to have the opinion of those interested on this subject. Atlanta extends her hospitality to all, and being a central point suggests itself as a desirable place of meeting.—ED.]

